# Entomological Profiles of Households in *Plasmodium falciparum* Case Foci and Comparison Areas in Grand’Anse, Haiti

**DOI:** 10.4269/ajtmh.24-0478

**Published:** 2025-02-25

**Authors:** Vena Joseph, Alice Sutcliffe, Laura Leite, Cyrille Czeher, Thomas Druetz, Eric Rogier, Thomas P. Eisele, Jean Frantz Lemoine, Michelle Chang, Daniel Impoinvil, Ruth A. Ashton

**Affiliations:** ^1^ Department of Social and Preventive Medicine, University of Montreal School of Public Health, Montreal, Canada;; ^2^ Division of Parasitic Diseases and Malaria, Centers for Disease Control and Prevention, Atlanta, Georgia;; ^3^ Institut de Recherche pour le Développement, Montpellier, France;; ^4^ Center for Applied Malaria Research and Evaluation, Tulane School of Public Health and Tropical Medicine, New Orleans, Louisiana;; ^5^ Ministry of Public Health and Population, Port-au-Prince, Haiti

## Abstract

Hispaniola, which is shared by Haiti and the Dominican Republic, remains the last island in the Caribbean that is still endemic for malaria, with Haiti bearing the highest caseload. Few studies have examined the ecology of malaria vectors in Haiti. Five species of *Anopheles* have been described on the island, but the exophilic *Anopheles albimanus* (*An. albimanus*) is considered the primary vector of malaria in Haiti. Households recruited for a case–control study profiling risk factors for symptomatic *Plasmodium falciparum* (*P. falciparum*) infections were approached to participate in an entomological study. The goal was to determine the bionomics of anopheline mosquitoes around the 32 participating households across varying malaria transmission settings. We assessed the characteristics of the *Anopheles* population using ultraviolet-light traps and larval surveys. *Anopheles albimanus* was the most abundant mosquito species identified in the Grand’Anse. Its abundance was higher in outdoor traps than in indoor traps and in areas with relatively high positivity based on rapid diagnostic test results. A greater proportion of blood-fed mosquitoes were found in higher transmission areas. *Anopheles albimanus* samples were found to be infected with both *P. falciparum* and *Plasmodium vivax* sporozoites. As Haiti aims for the elimination of malaria, disrupting localized residual malaria transmission will increasingly rely on focal vector control strategies.

## INTRODUCTION

At the time of this study in 2018, Haiti was progressing toward its goal of malaria elimination, and the transmission of the disease was becoming increasingly focal. In 2017, the estimated prevalence of malaria in Haiti ranged from <0.1 to 1%, with the highest incidence rates reported in the Grand’Anse Department. In 2017, Grand’Anse accounted for 54% of all malaria cases detected nationwide.^1^^,^^2^ Since then, progress toward malaria elimination has stagnated. The countrywide prevalence of malaria was 5.4% in 2023, with 52% of total malaria cases detected in the Grand’Anse Department (National Malaria Control Program, unpublished data).

The country’s national strategic plan aimed to eliminate malaria across its territory by 2025.^3^ To achieve this, the national malaria control program focused on several malaria elimination strategies including targeted or focal vector control, improved surveillance, and access to treatment through community case management. These routine interventions were occasionally supplemented with more aggressive parasite elimination strategies in areas showing both increased risk and high receptivity for malaria transmission (Haiti National Strategic Plan, unpublished).

Although the malaria eradication efforts on the island in the 1960s led to significant reductions in malaria prevalence in both Haiti and the Dominican Republic, these gains were not sustained in the long term. The 1980s saw a surge in morbidity and mortality in Haiti as funding for malaria control efforts waned.^4^ Since the establishment of the Haitian national malaria control program in 2005, there has been renewed interest in and funding for malaria elimination efforts.^3^^,^^5^^,^^6^ In addition to routine programmatic anti-malarial efforts,^7^ more intensive and large-scale campaigns have been deployed to target hotspots of infection.^8^ However, current malaria control efforts should take stock of the lessons from the past. The failure of past malaria elimination campaigns in Haiti has been attributed to limited resources, poor programmatic planning, lack of data on malaria transmission, dependence on external actors, and unsuccessful vector control strategies.^4^^,^^6^ These limitations must be addressed to ensure the success of the current strategic plan. Notably, the use of adequate vector control methods is necessary to disrupt localized, residual transmission and improve the effectiveness of elimination strategies.

Vector control methods should be specific to the population dynamics of the *Anopheles* species present in the targeted areas to ensure maximum efficiency of the deployed interventions.^9^^,^^10^ The use of site-specific and effective vector control is particularly crucial in resource-limited settings, such as Haiti, to achieve the goal of elimination and sustain those gains. However, the literature on anopheline mosquito populations in Haiti is limited, with the latest published works on the bionomics of anophelines in the country dating back to the 1990s.^11^ Since 1998, only four studies have been published on entomology in Haiti,^5^^,^^12^^–^^14^ none of which examine the vector populations in Grand’Anse. A systematic review of entomological studies in Haiti highlighted the lack of recent entomological data, with specific research gaps noted regarding the preferred indoor or outdoor biting locations of *Anopheles albimanus* (*An. albimanus*), peak biting times, and whether transmission occurs at elevations higher than 500 m.^5^

Five species of *Anopheles* have been described on the island: *An. albimanus*, *Anopheles grabhamii*, *Anopheles vestitipennis*, *Anopheles crucians*, and* Anopheles pseudopunctipennis*.^11^^,^^15^ However, *An. albimanus* is considered the primary vector of malaria in Haiti and is the only species thus far incriminated in malaria transmission.^5^

This study aimed to characterize the *An. albimanus* population in Grand’Anse, Haiti, by providing descriptive data on adult vector abundance and larval development sites in areas with relatively higher and lower *Plasmodium falciparum* (*P. falciparum*) burden. In addition, the species composition of local anopheline populations, blood meal sources, and sporozoite positivity are described. The entomological data presented here complement previously published data on the risk factors for malaria transmission in Grand’Anse.^16^ By providing data on the entomological risk factors for malaria, we also address some knowledge gaps regarding vector bionomics in Grand’Anse.

## MATERIALS AND METHODS

### Study area.

This study was conducted in the Anse-d’Hainault Arrondissement (or District) of the Grand’Anse Department in Haiti. The Grand’Anse Department lies on the southwestern peninsula of Haiti and is the country’s most forested department, characterized by mostly mountainous terrain. It is subject to trade winds blowing south to east and north to west throughout the year. Temperatures range from 25°C to 35°C in the coastal plains, with lower temperatures in the inland areas at higher elevations. The typical rainy season in Grand’Anse has two peaks during the year, one in May and one in October, with average monthly precipitations of 150 mm and 196 mm, respectively. The mean 30-day precipitation over the study area ranged from 79 to 140 mm for the duration of the surveys.^17^ The highly heterogeneous terrain and the perpendicular alignment of mountainous chains with respect to wind direction contribute to varied local precipitation patterns. Despite regional averages, rainfall does not reach all areas; mountain peaks and windward mountainsides receive the most rain, whereas valleys and leeward slopes are more arid. According to 2015 estimates, the Arrondissement of Anse-d’Hainault had a population of 98,522 people, the majority of whom live in rural settings.^18^ The elevations in the Anse-d’Hainault Arrondissement reach up to 600 m above sea level, and the primary activities include agriculture and fishing.^19^ Anse-d’Hainault was affected by Hurricane Matthew in 2016, and most of the local population had not yet recovered from the storm’s damages at the time of the study. Many of the homes in the communities visited for this study had not yet repaired the damage sustained during the hurricane. Some families were still living in temporary homes built after the storm, with roofs and parts of their walls made of plastic tarpaulin and iron sheets.

### Site selection.

A case–control study seeking to identify risk factors for *P. falciparum* infection was conducted between April and August 2018. Participants were enrolled from the population of patients seeking treatment for fever at four health facilities across the Anse-d’Hainault Arrondissement. Cases and controls (as determined using rapid diagnostic tests [RDTs]) were enrolled at the health facilities, and follow-up interviews were conducted at the participants’ homes. The full case–control study methods and results are described in Ashton et al.^16^ The participants from this case–control study^16^ were used as the pool from which households were recruited for the entomological data collection in this study.

**Figure 1.**
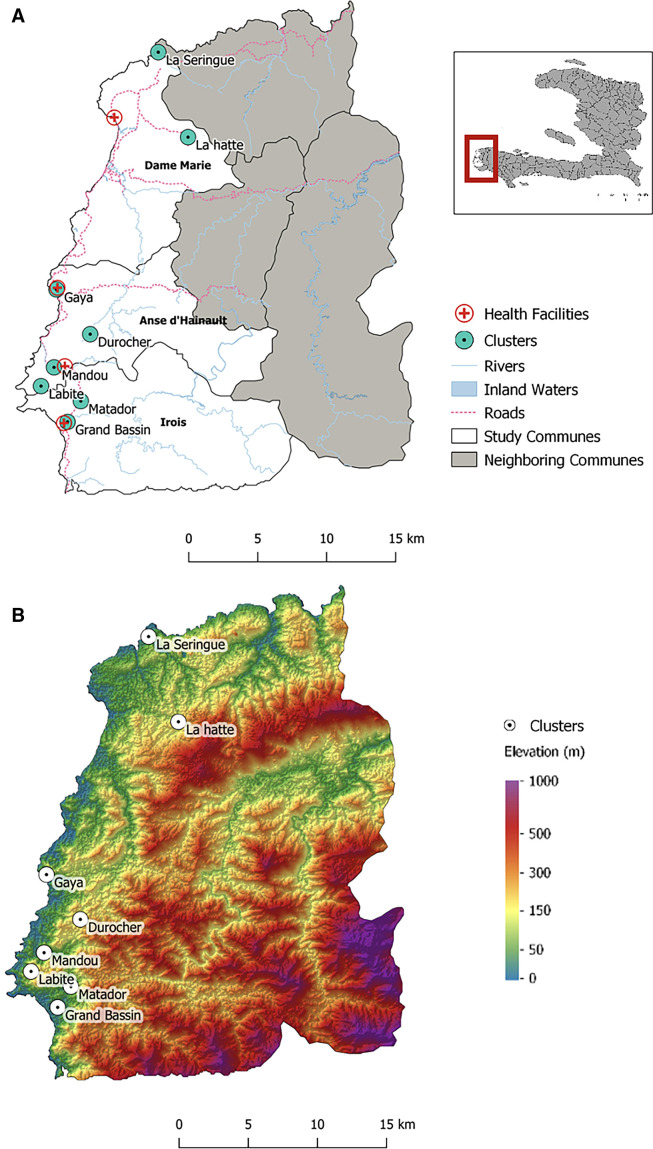
Map of the locations of sampling clusters and health facilities (**A**) and the corresponding topographical map derived from a digital elevation model (**B**).

Eight clusters were purposefully selected for entomological trapping using the GPS coordinates of households recruited from the case–control study (Figure 1). Four clusters were drawn from locations that appeared to be *P. falciparum* transmission foci (i.e., where multiple RDT-positive cases had been identified in the case–control study), and four clusters were selected from locations that had only contributed RDT-negative participants. Clusters were defined as groups of houses within a 500 m radius. The identification of selected clusters was achieved by periodically assessing the household location of participants recruited for the case–control study. We used Kuldorff’s spatial scan statistic in SaTScan version 9.6 (Information Management Services Inc., Calverton, MD) to identify clusters of cases and clusters of control households.^16^^,^^20^ Four households within each cluster were approached to participate in entomological sampling on the basis of convenience sampling. If, within a given 500 m radius, fewer than four households had participated in the overarching case–control study, we recruited additional households for the entomological trapping. No malaria diagnostic tests were conducted for these additional households, and no malaria risk factor data were obtained. Because of logistical constraints, entomological data collection was conducted sequentially from cluster to cluster.

The clusters were classified as “case” or “control” according to participant recruitment into the epidemiological case–control study at the time of mosquito trapping. However, in two of the four control clusters, RDT-positive individuals were recruited for the epidemiological study after mosquito trapping was completed. After reviewing the ratio of RDT-positive to RDT-negative participants recruited in the epidemiological study from the eight mosquito trapping clusters, the original case–control clusters were retained because “case” clusters continued to reflect areas with a high malaria burden, whereas “control” clusters represented areas with a relatively low burden (Table 1).

**Table 1:** Characteristics of trapping clusters

Cluster	Classification	Survey Period	RDT Pos. (Pos/Neg)*	Mean NDVI	Elevation (asl)	Land Surface Temperature	% Catches with >0 Aa Caught^†^
Control clusters							
Mandou	Peri-urban	May 18–22	4% (1/24)	167	45–100 m	28.0°C	65%
Lahatte	Rural	July 20–23	0% (0/7)	170	99–130 m	29.5°C	9%
Gaya	Urban	August 2–6	3% (4/114)	151	0–31 m	30.1°C	60%
Durocher	Rural	August 11–14	0% (0/15)	160	131–157 m	24.7°C	84%
Case clusters							
Labite	Rural	May 31–June 4	54% (33/28)	165	161–227 m	29.2°C	95%
Grand Bassin	Urban	June 9–13	26% (25/73)	160	0–12 m	23.1°C	91%
Matador	Peri-urban	July 22–14	17% (5/26)	160	4–38 m	30.9°C	97%
Laseringue	Rural	July 27–30	53% (19/17)	162	0–46 m	25.9°C	34%

Aa = *Anopheles albimanus*; asl = above sea level; NDVI = Normalized Difference Vegetation Index; Neg = negative; Pos = positive; RDT = rapid diagnostic test.

* Note that the total RDT results reflects individuals identified in the epidemiological study both before and after mosquito trapping had occurred.

^†^ Number of nights mosquitoes were caught/total number of trap nights.

All households with an adult member present at the time of enrollment in the study consented to participate. In cases in which no adult was available to provide consent, the selected household was replaced with the nearest house that had a consenting adult present. Local community members were eager to support the mosquito trapping efforts and accompany the larval development site surveys. Household members indicated where waterbodies typically formed during the rainy season whenever waterbodies could not be identified within the clusters.

### Survey of larval sites.

The surroundings of the houses selected for entomological data collection were inspected for the presence of any potential natural or man-made larval development sites within a radius of ∼250 m. This radius was chosen to ensure that all potential larval breeding sites near a household could be surveyed by a single data collector in a timely manner. Any identified waterbodies were geo-located using a hand-held GPS device. The potential larval development sites were described and classified according to sun exposure, size, salinity, turbidity, presence of algae, fish, debris, and the use of the water by the community. The larval development sites were inspected for the presence of any mosquito larvae (anopheline or culicine) using a standard dipper consisting of a 300 mL cup attached to a 3-foot-long stick. On average, six dips were taken per waterbody. The number of dips was adjusted on the basis of the surface area of the sampled waterbodies, with six additional dips taken for every additional meter of the circumference of the potential breeding site. The presence and number of anophelines or culicines were recorded on a data collection form. No larval specimens were retained, as the time and resources required to store, transport, and process the larval samples exceeded those available for this study.

### Adult mosquito collections.

Following the identification of a cluster, members of the selected houses were approached and asked to participate in the entomological study. Once verbal consent was obtained from the head of the household, two ultraviolet (UV) light traps (The New Standard Miniature BlackLight [UV] Trap Model 1212, John W. Hock Company, Gainesville, FL) were placed in each house: one inside, close to a sleeping space where the occupants slept under a bed net, and one outdoors, within 2 meters of the house, in a location shielded from rain and strong winds. The UV light traps were used to estimate the host-seeking behavior of the mosquito population. Untreated bed nets were provided to household members who did not have a net at the time of trapping; however, the bed nets already owned by household members were not replaced. Traps were positioned so that the bottom of the collection cage hung ∼1.5 m from the ground. The traps were set and activated at sunset each night (between 6:30 pm and 7:30 pm) and operated until sunrise (between 5:30 am and 6:30 am) the following morning, after which they were retrieved. For each house, the UV light traps were set for 3–5 consecutive nights to account for nightly fluctuations in mosquito presence. Mosquito trapping was conducted between May 18, 2018 and August 11, 2018.

### Morphological identification of adult mosquitoes.

Collection containers were removed from the UV light traps each morning, placed on ice to immobilize insects, and transferred to Petri dishes for the morphological identification of female mosquitoes. Insects other than mosquitoes were removed, and the remaining mosquitoes were sorted by genus (*Anopheles*, *Culex*, or *Aedes*). When possible, *An. albimanus* mosquitoes were distinguished from non-*An. albimanus* anophelines. Given the time and logistical constraints for processing the mosquitoes in the field and laboratory, we prioritized the female mosquitoes, which are directly involved in malaria transmission. Consequently, all male mosquitoes were discarded. Female *An. albimanus* were differentiated from non-*An. albimanus* species through morphological identification using a modified key based on Desenfant 1988.^15^ Female *Anopheles* specimens were stored individually in 1.7 mL Eppendorf tubes with silica beads, whereas culicine mosquitoes were stored in pools of up to 50 mosquitoes. All mosquito specimens were kept at ambient temperature.

### Mosquito dissection and grinding.

Collected female *Anopheles* mosquito specimens were maintained at ambient temperature until arrival at the CDC in Atlanta, GA, where they were stored at 4°C. Legs and wings were removed from the mosquitoes, and the head-thoraces were bisected from the abdomens using a scalpel following the method described by Foley et al.^21^ Head-thoraces were stored at –20°C until ∼1 day before being processed using a sporozoite bead assay. Abdomens that visibly contained bloodmeal were used for DNA extraction and polymerase chain reaction (PCR) analysis. The remaining body parts were returned to the original collection tube and stored at 4°C. Head-thoraces were homogenized in grind buffer (0.5% w/v casein, 0.002% w/v phenol red in 10 nM phosphate-buffered saline [PBS]; pH 7.4) and centrifuged to separate out liquid and debris. A laboratory colony of uninfected adult *An. gambiae* G3 mosquitoes, maintained at the CDC, were used as negative controls in the bead assay. Previously blood-fed mosquitoes were used as controls for the bloodmeal analysis. The control mosquitoes were processed separately, following the same procedure as the field-collected mosquitoes.

### Sporozoite detection via multiplex bead assay.

*Plasmodium* sporozoite detection was conducted on the processed mosquito samples using a circumsporozoite multiplex bead assay (csMBA), as described in Sutcliffe et al.^22^ Lyophilized positive controls and monoclonal antibodies specific for *P. falciparum*, *Plasmodium vivax* (*P. vivax*) VK210 (Pv210), and *P. vivax* VK247 (Pv247) circumsporozoite (cs) proteins were obtained from BEI Resources, National Institute of Allergy and Infectious Diseases, NIH (MRA-890, MRA-1028K). The *P. falciparum*, Pv210, and Pv247 monoclonal antibodies were covalently bound to polystyrene Bio-Plex^®^ COOH beads (Bio-Rad, Hercules, CA; 1715060XX) using the Luminex^®^ xMAP^®^ Antibody Coupling Kit (Luminex, Austin, TX; 40-50016) according to the manufacturer’s protocols. Detection antibodies were prepared by biotinylating the *P. falciparum*, Pv2010, and Pv247 monoclonal antibodies, each in a separate reaction, using the Thermo Fisher Scientific EZ-Link Micro Sulfo-NHS-Biotinylation Kit (Thermo Fisher Scientific, Waltham, MA; 21217) according to the manufacturer’s protocol.

All antibody assays were multiplexed using *P. falciparum*, Pv210, and Pv247 coupled beads and detection antibodies. The mosquito samples were incubated sequentially with the coupled beads, biotinylated detection antibody, and streptavidin-phycoerythrin (Invitrogen, Waltham, MA; 2866). The incubations were performed in 96-well plates at room temperature (∼20°C), protected from light, on a plate shaker (IKA, Staufen, Germany; microtiter shaker model 2/4) at 900 rpm. Three wash steps with 10 mM PBS, 0.05% Tween 20 were performed between each incubation. The non-magnetic polystyrene beads were washed using a vacuum. The samples were incubated with the coupled beads for 1.5 hours, then with the biotinylated detection antibody for 45 minutes, and finally with streptavidin-phycoerythrin for 30 minutes. A final incubation was conducted with reagent diluent (0.45 µM filter-sterilized 10mM phosphate-buffered saline, 0.05% Tween 20, 0.5% Bovine serum albumin) for 30 minutes before resuspension in 100 µL of PBS for a brief (1–2 minute) incubation.

The incubated sample plates were analyzed using a Bio-Plex^®^ 200 system (Bio-Rad; 171000201) with the Bio-Plex^®^ Manager™ software v6.2 (Bio-Rad). This system detects the fluorescence emitted from the microspheres and the phycoerythrin-conjugated detection antibody, reporting the median fluorescence intensity (MFI). The final assay signal is calculated as the difference between the average MFI values in sample wells and the average background (bkgd) values (MFI-bkgd) from wells containing only circumsporozoite enzyme-linked immunosorbent assay grind buffer. Samples with an MFI-bkgd value of ≥100 were classified as positive for cs protein, whereas those with an MFI-bkgd value of <100 were considered negative. Samples that initially tested positive (“initial test”) were boiled for 10 minutes at 100°C to denature any heat-unstable cross-reactive proteins.^23^ The sample was then retested (“boiled retest”) using csMBA. Both a positive initial test result and a positive boiled retest result were required for a sample to be considered positive. A detailed description of the laboratory procedures is available in Supplemental Material 1.

### Molecular identification of *Anopheles* species and their bloodmeal hosts.

A molecular analysis was conducted on the abdomens, wings, and legs of mosquitoes whose species could not be determined through morphological identification alone. To identify the *Anopheles* species, we used a single-step PCR^24^ targeting a fragment of the second internal transcribed spacer region (ITS2) in anophelines. All reactions containing a fragment of ∼500 base pairs were prepared for sequencing using the ITS2A and ITS2B primers. DNA extractions from wings and legs were performed using a previously described cetyltrimethylammonium bromide-based method for mosquitoes.^25^

A molecular analysis was performed on the abdomens of mosquitoes visually identified as blood-fed, to determine the source of the bloodmeal. DNA extractions from the abdomens were performed using DNAzol™ BD Reagent for the isolation of genomic DNA from whole blood (Thermo Fisher Scientific; 10974020). The DNAzol protocol was followed using half the volume of reagents recommended to account for the smaller volume of blood in the mosquito samples. Bloodmeal host species were analyzed using a hemi-nested PCR^26^ targeting a fragment of mammalian 16S mitochondrial ribosomal DNA. All second-round reactions containing a fragment of ∼500 base pairs were prepared for sequencing using the Mammalian-F and Mammalian-R primers.

All PCRs were performed in 25 µL reactions, as described in Supplemental Table 1. Selected reactions were prepared for sequencing using ExoSAP-IT™ (Thermo Fisher Scientific; 78-205-10M) according to the manufacturer’s directions. Sequencing was performed with the BigDye Terminator Ready Reaction Kit v1.1 and ABI 3500XL Genetic Analyzer (Applied Biosystems, Foster City, CA). Sequences were compared with DNA nucleotide sequences in National Center for Biotechnology Information basic local alignment search tool (BLAST) using the nucleotide BLAST query. The criteria for reporting a match were results achieving a ≥98% match and expect values ≤0.05.

### Remote sensing data.

Daily land surface temperatures were obtained from the Terra Moderate Resolution Imaging Spectroradiometer (MODIS) Land Surface Temperature/Emissivity Daily (MOD11A1) Version 6.1 (USGS, Washington, DC). The land surface temperature data has a spatial resolution of 1 km.^27^ The daytime land surface temperature was averaged over the days of trapping at each cluster. We used the 16-day Terra MODIS Normalized Difference Vegetation Index (MOD13Q1) data to measure vegetation cover in the sampling areas.^28^ Rainfall data were obtained from the Climate Hazards Group InfraRed Precipitation with Station data.^17^ We extracted the daily rainfall estimate for each site between April 1, 2018 and August 15, 2018. The 2-week accumulated rainfall was determined by summing the daily rainfall estimates for the 15 days preceding the first night of trapping at each household. Digital elevation data were obtained from the Shuttle Radar Topography Mission at the Earth Resources Observation and Science Center with a resolution of 90 m.

## STATISTICAL ANALYSES

Statistical analyses were conducted in R version 4.2.2 (R Foundation for Statistical Computing, Vienna, Austria), and maps were generated in qGIS version 3.28 (qGIS, London, United Kingdom). Summary statistics were generated to describe the characteristics of all potential larval breeding sites. We generated descriptive summaries for *Anopheles* specimens collected by location (trap placement, household identification, cluster identification, and transmission level), species, blood-fed status, and source of bloodmeal. For each household, the total number of mosquitoes caught per trap location was used to calculate the household-level outdoor-to-indoor ratio. This resulted in a total of 288 observations, each representing the number of mosquitoes found per location (outdoor or indoor trap placement), per night, for a given household. Average values and standard errors were computed from the arithmetic means of total household-level values for the three variables of interest (mosquitoes caught in the outdoor and indoor traps, and the ratio of outdoor-to-indoor mosquitoes). Summary statistics (mean and standard error) were computed at the cluster level (*n* = 8) or the malaria transmission level (case versus control). Due to the dispersion in the values for our outcome data, we used Mann–Whitney–Wilcoxon and Wilcoxon rank sum tests to assess differences in mosquito abundance between indoor and outdoor trap locations, as well as between-cluster classifications. A χ^2^ test of independence was used where appropriate to test the association between the presence of blood-fed and infected mosquito samples and the cluster classification of the trapping household. In cases in which expected values were less than five, Fisher’s exact test was performed to assess independence.

## RESULTS

### Cluster characteristics.

**Figure 2.**
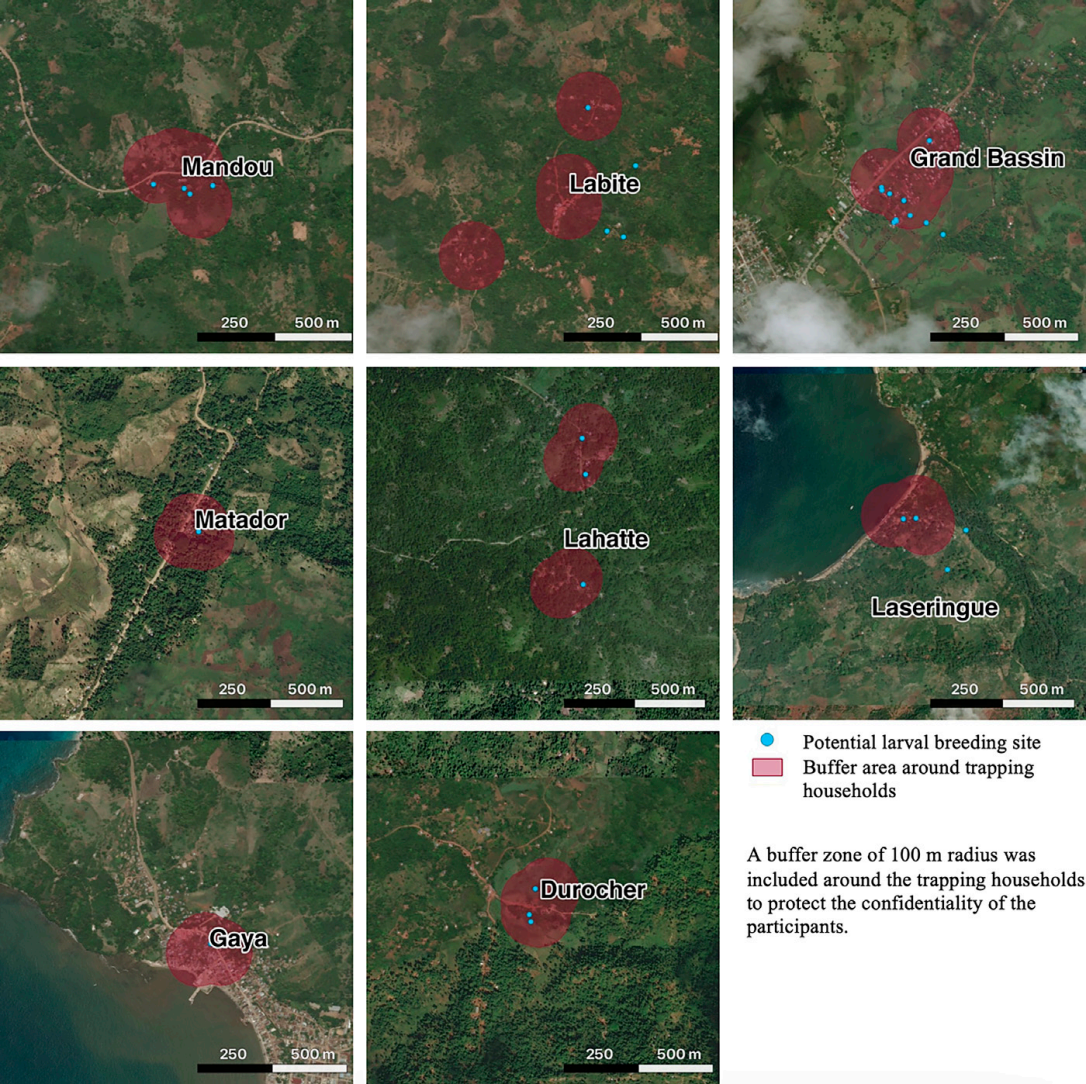
Satellite imagery of the eight sampling clusters and the location of the breeding sites. Images obtained from Bing Aerial Maps for the year 2018.

Four houses in each of the eight clusters were selected to participate in the entomological collections based on the location of participants recruited for the main case–control study. We hypothesized that the vector populations would be similar among households within a given cluster. Sampling sites were chosen to represent a variety of environmental settings, including varying proximity to the main road, vegetation coverage, elevation, and distance from the coast. Most trapping clusters were located along the coast of the Grand’Anse peninsula, where population settlements are clustered closely together. Some clusters were relatively far from the main road (Labite, Lahatte, Laseringue) and difficult to access. Although most clusters were near the coast, Durocher and Lahatte were more inland and at significantly higher elevations than the other clusters (Figure 1). Some clusters were in urban and peri-urban areas (Grand Bassin, Gaya) with concentrated settlement patterns and lower tree coverage, whereas others were in rural settings with higher tree coverage (Durocher, Matador) and more dispersed settlement patterns (Lahatte, Labite; Figure 2).

### Characterization of larval development.

Of the 33 waterbodies identified as potential larval breeding sites, 21 were located in the case clusters (the areas with a relatively higher malaria burden). The average distance from households to the closest breeding sites was lower in the case clusters (44 m* ±* 1.5) compared with the control clusters (77 m* ±* 0.4). Overall, 26% of waterbodies were positive for *Anopheles* larvae, whereas 52% were positive for culicine larvae. *Anopheles* larvae were primarily found in partially shaded, fresh, clear, and cloudy waterbodies (Table 2), accompanied by a variety of vegetation types (see Supplemental Figure 1). The habitats that tested positive for *Anopheles* larvae included rivers, lagoons, puddles, and man-made structures, such as canals and concrete reservoirs. Supplemental Figure 1 shows photos of a subset of larval sites identified in the study area. The most abundant habitat types around the participating households were rivers and puddles of varying sizes.

**Table 2:** Characteristics of the potential and active larval development sites surveyed

Characteristics of All Larval Sites		Control Clusters			Case Clusters			Total Larval Sites Surveyed		
		% Anopheles- Positive (*n*)	% Culicine-Positive (*n*)	*N*	% Anopheles- Positive (*n*)	% Culicine-Positive (*n*)	*N*	% Anopheles- Positive (*n*)	% Culicine-Positive (*n*)	*N*
Salinity	Fresh	16.67% (2)	25% (3)	12	25% (5)	65% (13)	20	21.87% (7)	50% (16)	32
	Brackish	0	0	0	100% (1)	0	1	100% (1)	0%	1
Turbidity	Clear	11.11% (1)	22.22% (2)	9	27.27% (3)	45.45% (5)	11	20.00% (4)	35.00% (7)	20
	Cloudy	33.33% (1)	33.33% (1)	3	30.00% (3)	80.0% (8)	10	30.77% (4)	69.23% (9)	13
Sun exposure	Sunlit	0%	33.33% (1)	3	0%%	71.43% (5)	7	0%	60.00% (6)	10
	Partial shade	28.57% (2)	28.57% (2)	7	42.86% (6)	57.14% (8)	14	38.09% (8)	47.62% (10)	21
	Total shade	0%	0%	2	0	0	0	0%	0%	2
Type	Permanent	18.18% (2)	27.27% (3)	11	50.00% (5)	60.00% (6)	10	33.33% (7)	42.86% (9)	21
	Temporary	0%	0%	1	9.09% (1)	63.63% (7)	11	8.33% (1)	58.33% (7)	12
Total		16.67% (2)	25% (3)	12	28.57% (6)	61.90% (13)	21	24.24% (8)	48.48% (16)	33

The numbers of larval sites are shown in parentheses (*n*). The proportion of larval development sites found positive for *Anopheles* and Culicine larvae are shown in the case and control clusters.

The percentage of larval sites found positive for *Anopheles* larvae varied from cluster to cluster (ranging from 3% to 36%), with the Grand Bassin cluster containing the highest percentage of positive larval sites, whereas Gaya and Matador contained the lowest percentage of positive larval sites (Supplemental Table 2). Overall, the percentage of larval sites found positive for *Anopheles* larvae was higher in the case clusters (28.6%; *n* = 6) compared with the controls (16.7%; *n* = 2). The same was true for larval sites positive for culicine larvae, with 61.9% (*n* = 13) of larval sites found positive for culicine larvae in the case clusters compared with 25% (*n* = 3) in the controls. Fisher’s exact test revealed that the difference between positive larval sites in case versus control clusters was not significant for both *Anopheles* (*P* = 0.68) and culicine larvae (*P* = 0.071).

### Adult mosquito species and their abundance by indoor and outdoor locations.

A total of 2,906 adult female mosquitoes were collected in UV light traps across the eight clusters. Of these, approximately half were morphologically identified as *An. albimanus* in the field (*n* = 1,443; 51.2%). A total of 1,375 (46%) specimens were culicine mosquitoes, including *Aedes* sp., *Culex* sp., and other *Culicinae*. Morphological methods identified a small number of anophelines (*n* = 58) as non-*An. albimanus* anophelines. Some mosquitoes (*n* = 30) could not be identified morphologically because of damage. The 88 unconfirmed mosquitoes, which included those morphologically identified as non-*An. albimanus* anophelines and the unidentifiable samples, were marked for species confirmation using single-step PCR on the ITS2 region. The ITS2 analysis was conducted on 74 samples, confirming 55 as *An. albimanus*. For 13 samples, no band could be amplified using a PCR analysis, and for six samples, no species match could be identified. We use the species confirmed by molecular analysis in our reports of mosquito species abundance for samples for which laboratory data were available. We only use the species reported after morphological identification for samples for which molecular identification could not be performed.

**Figure 3.**
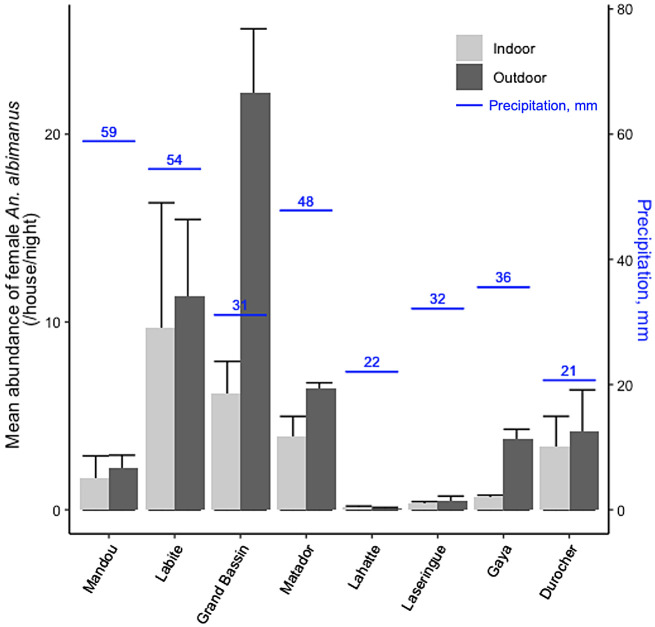
Mean abundance of female *Anopheles albimanus* adults and accumulated biweekly rainfall (mm) in the eight trapping clusters. The error bars show the standard errors in the mean mosquito abundance for each cluster.

Cumulative rainfall in the 15 days before the start of mosquito trapping was variable and did not clearly correspond with mosquito abundance (Figure 3). The final four clusters sampled (Lahatte, Laseringue, Gaya, and Durocher) had comparable rainfall leading up to the days of trapping, yet exhibited different mosquito populations. This indicates that the presence of larval sites around the clusters would serve as a better indicator of mosquito population dynamics than total rainfall in the area.

The abundance of *An. albimanus* mosquitoes, measured by the average number of female *An. albimanus* caught per house per night, was higher outdoors at six of the eight sites. Conversely, culicine mosquitoes were predominantly found indoors. Overall, the outdoor-to-indoor ratio of *An. albimanus* caught in traps across all trapping clusters was 2.99 (±0.51), compared with 1.27 (±0.52) for culicines. The ratio of outdoor-to-indoor *Anopheles* catches was even higher in clusters located in more concentrated settlement patterns, such as Gaya and Grand Bassin (Table 3).

**Table 3:** Mean abundance of female *Anopheles albimanus* adults per house per trapping night and ratio of outdoor-to-indoor catches across the eight clusters

Cluster	*An. albimanus*			Culicinae		
	Mosquitoes/House/Night		Ratio Outdoor-to-Indoor	Mosquitoes/House/Night		Ratio Outdoor-to-Indoor
	Outdoor	Indoor		Outdoor	Indoor	
Control clusters	2.56 (0.67)	1.46 (0.55)	3.37 (0.80)	3.32 (0.95)	9.12 (2.67)	0.41 (0.078)
Mandou	2.25 (0.66)	1.70 (1.17)	2.76 (0.88)	0.90 (0.21)	2.45 (0.61)	0.42 (0.125)
Lahatte	0.06 (0.06)	0.12 (0.72)	0.50 (0.35)	0.19(0.120)	0.56 (0.157)	0.38 (0.239)
Gaya	3.75 (0.54)	0.65 (0.13)	6.52 (1.86)	8.30 (1.43)	13.85 (1.93)	0.64 (0.131)
Durocher	4.19 (2.20)	3.38 (1.60)	2.28 (1.25)	3.88 (1.53)	19.62 (7.43)	0.19 (0.019)
Case clusters	10.1 (2.38)	5.04 (1.79)	2.62 (0.65)	3.13 (0.64)	4.23 (1.18)	2.13 (1.01)
Labite	11.4 (4.11)	9.70 (6.65)	3.23 (2.23)	0.85 (0.25)	1.55 (0.42)	0.74 (0.273)
Grand Bassin	22.2 (3.41)	6.20 (1.70)	4.04 (0.75)	1.95 (0.43)	4.35 (0.94)	0.59 (0.220)
Matador	6.47 (0.29)	3.93 (1.04)	2.07 (0.55)	3.90 (1.42)	8.40 (4.07)	0.81 (0.405)
Laseringue	0.50 (0.23)	0.31 (0.12)	0.67 (0.38)	5.81 (1.02)	2.62 (1.17)	6.36 (3.49)
Total	6.35 (1.39)	3.25 (0.97)	2.99 (0.51)	3.22 (0.56)	6.68 (1.50)	1.27 (0.52)

For each household, the number of *An. albimanus* and culicine mosquitoes caught in each trap location is used to calculate the household-level outdoor-to-indoor ratio for each trapping night. The cluster level value for the outdoor-to-indoor ratio is reported as the average of these individual household ratios. This method allows the individual household dynamics to skew the mean, providing information on the overall dispersion of the mosquito populations in individual households for each cluster. The standard errors are given in parentheses.

Cluster classification of the trapping sites based on RDT positivity indicated that the total number of vectors caught per household was higher in the case clusters compared with the controls. The average number of anopheline mosquitoes caught indoors was greater in the case clusters (5.04 ± 1.79) than in the control clusters (1.46 ± 0.55). The ratio of outdoor-to-indoor *An. albimanus* was smaller in the case clusters (2.62 ± 0.65) compared with the control clusters (3.37 ± 0.80). Conversely, when comparing the ratio of culicines between case and control areas, culicine mosquitoes constituted a higher proportion of mosquitoes caught in the control clusters compared with the cases. The ratio of outdoor-to-indoor culicines was higher in the case clusters (2.12 ± 1.01) than in the control clusters (0.405 ± 0.078).

**Figure 4.**
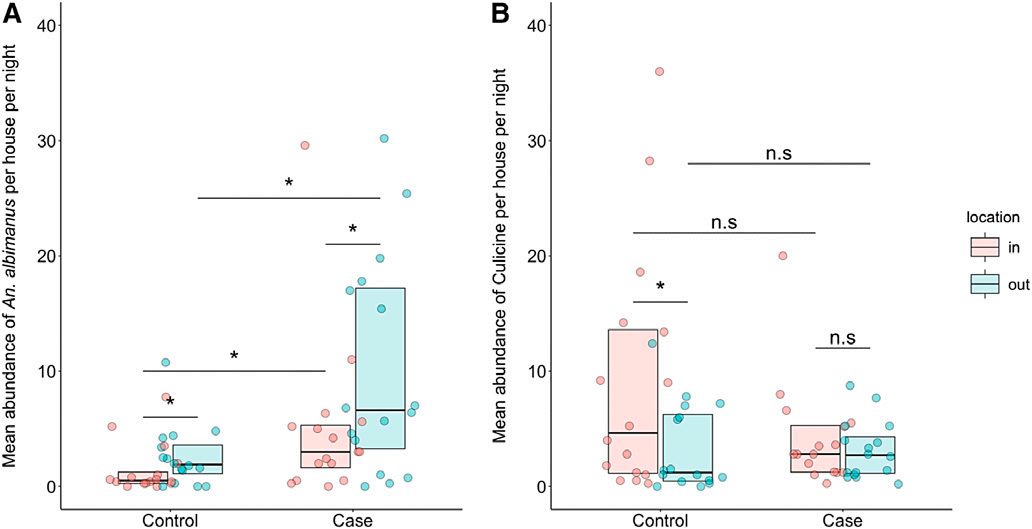
A comparison of the mean abundance of *Anopheles albimanus* (**A**) and culicine (**B**) mosquitoes according to the cluster classification of the house and trap location. The individual points represent the mean mosquitoes caught per house per night observations at each sampling location. The three horizontal lines of the box plots show the 25th, 50th, and 75th percentiles, respectively. *P <0.05, the *P*-value yield by a Mann–Whitney–Wilcoxon test (for comparisons between case and control) or a Wilcoxon rank sign test for comparison of indoor vs. outdoor abundance within a cluster category. n.s = not significant.

The results of a Mann–Whitney–Wilcoxon test indicate that the abundance of *An. albimanus* was significantly higher in case clusters compared with control clusters for both outdoor-caught mosquitoes (*P* = 0.012) and indoor-caught mosquitoes (*P* = 0.022). However, the difference in culicine mosquito abundance between case and control clusters was not significant, regardless of trap location (Figure 4). A Wilcoxon rank sign test showed that the average abundance of *An. albimanus* was significantly higher for outdoor catches compared with indoor catches in both case clusters (*P* = 0.0076) and control clusters (*P* = 0.017). In contrast, the average abundance of adult culicine mosquitoes was significantly higher indoors compared with outdoors in the control clusters (*P* = 0.00072). However, no significant difference was detected in the average abundance of culicine mosquitoes caught indoors compared with those caught outdoors in the case clusters (*P* = 0.43). There was no significant difference in the outdoor-to-indoor ratio of *An. albimanus* between the control and case clusters (*P* = 0.60). However, the outdoor-to-indoor ratio of culicines was significantly lower in the control clusters (*P* = 0.026).

### *Anopheles* blood meal sources and sporozoite rates.

A total of 126 *Anopheles* mosquitoes caught in the light traps were blood-fed. For 75 (59.5%) of those samples, the source of the blood meal could be determined. The most common bloodmeal source was humans (*n* = 29); other blood sources included cows (*n* = 7), sheep (*n* = 5), pigs (*n* = 5), horses (*n* = 3), donkeys (*n* = 1), and dogs (*n* = 1). Blood-fed *An. albimanus* accounted for 8.9% of all anophelines caught in the case clusters, compared with 2.4% of anophelines in the control clusters. A χ^2^ test showed a significant difference in the proportion of blood-fed *An. albimanus* between case and control clusters (*P* = 0.011).

Of the 1,737 *Anopheles* tested using bead assay csMBA, 22 samples were infected with *Plasmodium* sporozoites (1.3%). Twenty of the sporozoite-positive samples were identified as *An. albimanus*, but two had no significant match in the ITS2 analysis, preventing the resolution of the mosquito species for those specimens. One of the two specimens with no match in the ITS2 database was morphologically identified as *An. albimanu*s, whereas the other specimen could not be identified morphologically. Most sporozoite-positive mosquitoes were collected from three case clusters (fourteen in Labite, six in Grand Bassin, and one in Matador), with the remainder from one control cluster (one sporozoite-positive mosquito in Gaya).

Among the 22 sporozoite-positive mosquitoes, 19 infections were identified as *P. vivax*, and 3 were identified as *P. falciparum* via bead assay*.* The two mosquitoes that could not be identified at the species level were determined to be positive for *P. falciparum*. Mosquitoes infected with *P. vivax* were collected from all four study clusters that contributed sporozoite-positive specimens. Fisher’s exact test on the sporozoite data indicated no association between case/control classification and the number of mosquito specimens found infected with *Plasmodium* sporozoites (*P* = 0.39).

## DISCUSSION

We present results on the bionomics of *An. albimanus* in areas of high and low RDT positivity for *P. falciparum* in Grand’Anse. *An. albimanus* was the single most abundant species caught in UV light traps, accounting for 54% of all 392 mosquitoes collected. It was also found to be positive for *P. falciparum and P. vivax.* No historical data are available regarding the bionomics of *An. albimanus* in Grand’Anse. It is unclear whether this species has always been dominant in Grand’Anse or if its population density has increased over time. Adult anophelines were more frequently caught outdoors than indoors, providing evidence of the exophilic behavior of the vector in Grand’Anse. These findings address important gaps in the current knowledge of the vector population in the region with the highest malaria burden in the country.

Our results suggest an association between the level of malaria transmission and the abundance of female anophelines. More female *An. albimanus* were caught in UV light traps placed in households in high-transmission areas (the case clusters) compared with those in low malaria transmission areas (control clusters). The abundance of *An. albimanus* was higher in the case clusters, regardless of the location of the UV light traps. This indicates that more female *An. albimanus* were caught in the indoor traps in case clusters compared with the indoor traps in control clusters. The same was true for outdoor traps. However, no significant difference was detected in the number of larval sites that tested positive for *An. albimanus* larvae between case and control clusters.

More blood-fed *An. albimanus* were found in case clusters compared with the control clusters, indicating that in addition to having higher mosquito abundance, case clusters are also characterized by higher biting rates. Most of the sporozoite-positive *An. albimanus* samples came from case clusters; however, no significant association could be demonstrated between sporozoite positivity and cluster transmission levels. Our findings illuminate potential avenues to enhance vector control as a strategy to reduce malaria transmission in Grand’Anse.

Haiti’s current vector control strategies use insecticide-treated nets (ITNs) as a primary intervention and deploy focal indoor residual spraying (IRS) in high-transmission areas as needed.^6^ The last IRS campaign in Grand’Anse occurred in November 2020, whereas the last ITN campaign was conducted in November 2023. The protective efficacy of bed nets against species with predominantly exophagic behaviors, such as *An. albimanus*, has been disputed^29^; however, recent analyses conducted by the Malaria Zero consortium show a decline in malaria incidence following a mass bed net distribution campaign in Southern Haiti (Eaton et al., in preparation). We found a significantly higher number of adult *An. albimanus* in case clusters compared with controls, regardless of trap location. This finding suggests that the use of bed nets may be an important intervention to curtail malaria transmission by preventing indoor biting during normal sleeping hours. Therefore, targeted ITN distributions in high-transmission areas should be an effective malaria control intervention. However, mass distribution campaigns on a large scale may not necessarily lead to reductions in malaria transmission. In any case, ITN campaigns should not be the only vector control strategy deployed in Grand’Anse. The inability to account for the exophilic and exophagic behaviors of some anopheline species is often cited as one cause for the failure of the original Global Malaria Elimination Program.^29^ This is an important consideration for the Grand’Anse Department. For vector control campaigns to be successful in this setting, they should incorporate interventions targeting the vectors outdoors. Therefore, more data is needed on the outdoor resting behavior of *An. albimanus* in high-transmission settings like Grand’Anse to better understand the impact of exophagic behavior on malaria transmission and to identify which interventions would be most appropriate to target those outdoor resting or biting vectors.

Given the exophilic behavior of *An. albimanus* in Grand’Anse, targeted ITN campaigns should be complemented with interventions focusing on outdoor transmission. Our study shows that anopheline mosquitoes were present in higher numbers in the outdoor traps across all transmission clusters, which was not the case for culicine mosquitoes. These findings provide evidence of the outdoor preference typically associated with this vector species, suggesting that targeted ITN alone will not be sufficient to stop malaria transmission. Furthermore, the possibility of behavioral resistance, or the emergence of population-wide behavioral changes in response to control strategies, should discourage the exclusive use of vector control strategies targeting indoor transmission. Indeed, the acclimation of *Anopheles* species in response to vector control methods has been documented in several countries.^30^ Reports have indicated behavioral shifts in naturally endophilic mosquito species due to IRS and net use, with some species shifting to more exophilic behavior and even changing host preferences.^31^^–^^33^ This suggests that relying exclusively on bed nets and IRS will not guarantee malaria elimination in Haiti. In fact, solely deploying IRS and ITN could further encourage exophilic behavior in a mosquito population that is already more prevalent outdoors. Areas with a high malaria burden may therefore benefit from interventions targeting outdoor residual transmission, such as larval control. Larval control could be implemented in select high- to moderate-transmission areas where ITN coverage is already high. Based on the study results, for example, Grand Bassin, Labite, and Laseringue would be targeted for larval control, given their high RDT positivity and the number of positive breeding sites identified around the clusters. In addition, improving access to diagnostics and treatment for malaria in high-transmission settings should remain a priority.

The detection of *P. vivax* sporozoites in *An. albimanus* specimens was unexpected. Even more surprising is the fact *that An. albimanus* sporozoite infection rates were higher for *P. vivax* compared with *P. falciparum*. Indeed, *P. falciparum* accounts for nearly 98% of all human infections in Haiti, whereas *P. vivax* consistently accounts for less than 1% based on microscopy, serology, and molecular techniques.^34^^–^^37^ One study suggested that the high frequency of the Duffy antigen-negative genotype in the Haitian population limits susceptibility to *P. vivax* infection.^38^ However, a possible reason for the higher detection of *P. vivax* in mosquitoes is that *An. albimanus* mosquitoes present in Haiti may be more susceptible to *P. vivax* infections than to *P. falciparum*. Three studies indicated that some* An. albimanus* population strains had higher infection rates with *P. vivax* than with *P. falciparum*.^39^^–^^41^ Although the high frequency of the Duffy antigen-negative genotype in the human Haitian population limits the spread of *P. vivax*, a higher infection rate with *P. vivax* in mosquitoes, coupled with the relatively high population density of *An. albimanus*, may increase the chance for *P. vivax* to propagate in the mosquito population, albeit at very low levels. Thus, even a few people with *P. vivax* infection may lead to several infected mosquitoes. Given the low occurrence of *P. vivax* infections in humans, it may be informative to assess the factors influencing this high detection of *P. vivax* in *An. albimanus* mosquitoes in Haiti to better understand malaria transmission dynamics. Alternatively, the multiplex bead assay used for sporozoite detection employed a stringent cutoff value (MFI-bkgd <100). This cutoff point for the cs assay has the potential to lead to false positives.^22^

There are several limitations to this study. First, trapping was conducted sequentially in the clusters. Although the results show variability in *An. albimanus* abundance between clusters, this variability could be partly attributable to temporal patterns rather than true between-cluster variation.^42^^,^^43^ Furthermore, additional environmental factors known to affect mosquito population dynamics differed for each sampling period, notably rainfall and temperature. The variability in these environmental factors may explain some of the differences in mosquito populations between clusters. Conducting the study concurrently in all clusters was not possible due to limited resources; however, long-term monitoring of mosquito populations across different sites using UV light traps should be considered in future studies. Some of the water bodies reported by the community members did not contain any water at the time of the study. One of the sites that local community members identified as an important breeding site for insects during the rainy season was a dry riverbed. Longitudinal studies of water bodies in these clusters may be necessary to understand the stability of larval breeding sites and their association with malaria transmission.

Another limitation of this study was the limited scope of the species identification efforts. We only provide data on adult female mosquitoes and did not retain adult males or larval specimens for species identification. Although we noted the number of culicine and anopheline larvae found in each waterbody, additional data on the species composition in the immature stages of the mosquitoes would have offered more insight into the population dynamics of the vector. Similarly, the species composition of the male mosquitoes would have contributed to a more comprehensive understanding of the vector population.

Finally, it is unclear how the distribution of untreated bed nets might have affected adult mosquito collections. Pyrethroid-treated nets can repel certain mosquitoes, leading to greater mosquito avoidance in households using treated nets compared with those with untreated nets. Although laboratory tests with *An. albimanus* in chambers showed the repelling effects of DDT, deltamethrin, and permethrin,^44^ it remains unclear how baiting treated versus untreated nets with a human host affects the capture of free-flying mosquitoes in light traps placed near beds. A study in Macha, Zambia, comparing *An. arabiensis* collection through human landing catches and CDC traps notes that ITNs did not appear to impact CDC trap effectiveness.^45^

In this study, the a priori selection of trapping clusters aimed to sample an equal number of case and control clusters across a wide range of land cover types and geographies given the time and resource constraints. The resulting data provide a preliminary profile of vector populations in these different settings; however, further studies are required to explain the spatial heterogeneity observed in vector densities across the regions. Furthermore, temporal changes in ecological and climatic factors may significantly impact the abundance of *An. albimanus*.^29^^,^^46^^–^^48^ More data are needed to better understand the population dynamics of the vector under changing environmental conditions. For example, future studies could explore how climatic changes due to global warming and physical alterations to the natural and man-made environments in Grand’Anse are likely to impact vector populations and, consequently, malaria transmission. Routine entomological monitoring should be reinforced in Grand’Anse to increase the quantity, quality, and granularity of the data on the structure of local mosquito communities. Additional information on the outdoor resting behavior of local anophelines is required to identify vector control strategies targeting outdoor biters. There is, of course, a cost associated with more intensive data collection. The willingness of local communities to participate in and support the mosquito trapping and larval surveys throughout this study suggests that participatory approaches to entomological monitoring are feasible.

## CONCLUSION

As Haiti moves toward malaria elimination, vector control efforts should be strengthened and tailored to the bionomics of local vector populations to maximize effectiveness. Additionally, further studies on the ecology of *Anopheles* species in Grand’Anse will be of paramount importance, not only to achieve the goal of malaria elimination but also to sustain those gains beyond 2025.

## Supplemental Materials

10.4269/ajtmh.24-0478 Supplemental Materials
