# Supplementary material for: Entomological Profiles of Households in Plasmodium falciparum Case Foci and Comparison Areas in Grand’Anse, Haiti
Source: Am J Trop Med Hyg. 2025 Feb 25;112(5):975–86. doi: 10.4269/ajtmh.24-0478 (PMC12062682; doi:10.4269/ajtmh.24-0478)
Supplement: Supplemental Materials [file tpmd240478.SD1.pdf]

Supplementary materials

1. Laboratory procedures

Mosquito dissection and grinding

Collected mosquitoes for laboratory testing were obtained from Grand'Anse, Haiti in 2018 and stored in 1.7ml Eppendorf tubes with silica beads at ambient temperature until arrival at the Centers for Disease Control and Prevention, Atlanta GA (CDC), where they were transferred to 4°C. A laboratory colony of uninfected adult *Anopheles gambiae* G3 mosquitoes, maintained in the CDC, were used as negative controls. Previously blood-fed control mosquitoes (5 to 10 days old) were placed in -20°C freezer overnight and then placed on Drierite® in a sealed container at room temperature (approximately 20°C) until use. Legs and wings were removed from field-collected and control mosquitoes, and the head-thoraces were bisected from abdomens using a scalpel according to Foley et al (2012). Each head-thorax was placed in a separate 1.2ml collection tube (Qiagen; 19560), containing a single 5mm stainless steel bead (Qiagen; 69989), arranged in 96-place tube racks. The remaining body parts were replaced in the original collection tube and stored at 4°C for later analysis. Head-thoraces were stored at -20°C approximately one day before being processed. One hundred microliters (100µl) of grind buffer (0.5% w/v casein, 0.002% w/v phenol red in 10mM phosphate-buffered saline, pH 7.4) was added to each tube and homogenized using a Qiagen TissueLyser II. Tube racks were placed in adapter sets (Qiagen; 69984) and agitated twice at 30Hz for 30 seconds, changing the orientation between each agitation. A brief centrifugation was performed to collect liquid and debris at the bottom of the tube and 150µl of grind buffer was added to each sample (total volume approx. 250 µl). Following testing, the remaining sample was transferred into a 96-well plate for storage at -20°C. Stainless steel beads were washed by soaking in 10% bleach, rinsing with pure water and, air-drying before re-use. Abdomens that visibly contained a bloodmeal were used for DNA extraction and PCR analysis.

MULTIPLEX BEAD ASSAY

Positive Controls and Antibodies

For the multiplex bead assay (MBA), lyophilized positive controls and monoclonal antibodies specific for *Plasmodium falciparum* (Pf), *P. vivax* VK210 (Pv210), and *P. vivax* VK247 (Pv247) circumsporozoite (cs) proteins, were obtained through BEI Resources, NIAID, NIH (MRA-890, MRA-1028K). All positive controls and antibodies were rehydrated to the concentrations indicated in the kit instructions (Malaria Research and Reference Reagent Resource Center) with blocking buffer (positive controls) or reverse-osmosis deionized water only (antibodies; no glycerol was added).

Binding of capture antibodies to polystyrene beads and biotinylation

Pf, Pv210, and Pv247 monoclonal antibodies were covalently bound to polystyrene Bio-Plex® COOH beads (Bio-Rad; 1715060XX), each with a different bead designation, using the Luminex® xMAP® Antibody Coupling Kit (Luminex; 40-50016) according to the manufacturer's protocols. All incubations were done in an auto-rotator at 30rpm at room temperature (approximately 20°C). Wash steps were performed using Activation Buffer (Luminex; 11-15171),

centrifuging for 1.5 minutes at 14548 x *g,* and vortexing at a low setting. Following removal of bead diluent and two washes, carboxyl groups on the microsphere surface were activated in a 20-minute incubation with the Activation Buffer, 1-Ethyl-3-(3-dimethylaminopropyl)carbodiimide (EDC) and N-hydroxysulfosuccinimide (Sulfo-NHS). Two additional wash steps were performed before beads were incubated for 2 hours with activation buffer and 10µg of monoclonal antibody per milliliter of beads, to allow carboxyl-to-antibody amine crosslinking. Two final washes were performed, and bead-coupled antibodies were resuspended in 1ml of Wash Buffer per 1ml of starting volume of beads (Luminex 11-251167) and stored at 4°C. A sufficient volume of beads was coupled to supply all experiments.

Pf, Pv210, and Pv247 monoclonal antibodies were biotinylated, each in a separate reaction, using ThermoScientific EZ-Link Micro Sulfo-NHS-Biotinylation Kit (ThermoScientific; 21217) according to the manufacturer's protocol. The sulfo-NHS-biotin concentration used for each coupling was 267.9µg per ml of antibody (originally resuspended to 0.5mg/ml. A sufficient volume of antibody was biotinylated for use across all experiments.

Circumsporozoite multiplex bead assay (csMBA) protocol

The circumsporozoite multiplex bead assay (csMBA) protocol was adapted from the Rogier et al (2017) antigen detection assay. All antibody assays were multiplexed containing Pf, Pv210, and Pv247 coupled beads and detection antibodies. Reagent diluent (0.45µM filter-sterilized 10mM phosphate-buffered saline, 0.05% tween 20, 0.5% BSA) was used to dilute bead-coupled antibody, biotinylated detection antibody and streptavidin-phycoerythrin (Invitrogen; 2866). In each step where reagent diluent was used, 50µl of the solution was applied per well. The 96-well plates with the samples were incubated at room temperature (approximately 20°C), protected from light, on a plate shaker (IKA; MTS 2/4) at 900rpm. Three wash steps with PBS-T (10mM phosphate-buffered saline, 0.05% Tween 20) were performed between each incubation. For the assay, filter bottom plates (Millipore; MADVN6550) were pre-wetted with PBS-tween and 50µl (approximately 1250 beads) of coupled beads were added, washed three times, and incubated with 50µl of sample for 1.5 hours. After washing, wells were incubated with a 50µl mixture of Pf, Pv210, and Pv247 biotinylated detection antibodies for 45 minutes. After washing, wells were incubated for 30-minutes with 50µl of streptavidin-phycoerythrin diluted at a ratio of 1:333 in reagent diluent (Invitrogen; 2866). A final incubation was done with reagent diluent for 30 minutes before resuspension in 100µl of PBS for a brief (1-2 minute) incubation.

Immediately following the resuspension of the sample in PBS and incubation, plates were either analyzed using a Bio-Plex® 200 system (BioRad; 171000201) with the Bio-Plex® Manager™ software v6.2 (Bio-Rad, USA) or stored at 4°C, protected from light, for up to 24 hours. Stored plates were washed three times following removal of PBS, wells were resuspended in 100µl of PBS using a brief (1-2 minute) incubation and then analyzed as described above (Zhang 2020, ThermoFisher Scientific). The Bio-Plex® 200 system detects emitted fluorescence from the microspheres and the phycoerythrin-conjugated detection antibody. Bio-Plex® Manager ™ software reports fluorescence as the median fluorescence intensity (MFI) for 50 beads and generates the mean MFI for replicates when applicable. Background (bkgd) values were determined by calculating the average MFI value of six wells on

each assay plate containing only csELISA grind buffer. The background value was subtracted from sample MFI values on respective plates to report the final assay signal as MFI-bkgd.

Samples with an MFI-bkgd value of ≥ 100 were classified as positive for cs protein and those with an MFI-bkgd value of < 100 were considered to be negative. A 50µl aliquot of the samples that initially tested positive ("initial test") was incubated in a thermal cycler (Bio-Rad T100) for 10 minutes at 100°C to denature any heat-unstable cross-reactive proteins (Durnez 2011). The sample was then retested ("boiled retest") by csMBA. A positive initial and boiled retest were both required for a sample to be considered positive.

Supplementary Table 1 Description of PCR assays

PCR Description		Primer sequences (5' to 3')	PCR reaction	Thermalcycling conditions	Agarose gel conditions
16S Bloodmeal host identification (Roellig 2013)	1st round	Vertebrate-F: ACC CNT CYM TGT NGC AAA AKR GTG<br>Mammalian-R: AYT GTC GAT AKG RAC TCT WRA RTA	1X Accustart II GelTrack Supermix (Quantabio; 95136), 0.4uM each primer, 1µl template DNA	94° for 2 min 30s; 40 cycles of 94°C for 30s, 55° for 30s, 72°C for 1 min	1% gel in TBE; 90 min at 85v
	2nd round	Mammalian-F: CCT GTT TAC CAA AAA CAT CAC<br>Mammalian-R: AYT GTC GAT AKG RAC TCT WRA RTA	1X Accustart II GelTrack Supermix, 0.4uM each primer, 1µl 1st round PCR product	94° for 2 min 30s; 35 cycles of 94°C for 30s, 52° for 30s, 72°C for 45s	1% gel in TBE; 90 min at 85v
ITS2 *Anopheles* species identification (Beebe 1995)		ITS2A: TGT GAA CTG CAG GAC ACA T<br>ITS2B: TAT GCT TAA ATT CAG GGG GT	1X PCR buffer, 80µM dNTP, 0.3mM $MgCl_2$, 0.5U GoTaq (Promega; M3001), 1µl template DNA	94° for 4 min; 35 cycles of 94°C for 60s, 51° for 60s, 72°C for 60s; 72°C for 10 min	1.5% gel in TBE; 60 min at 100v

Supplementary Table 2. Types of potential larval breeding sites surveyed in the 8 clusters.

	% potential larval breeding sites found in each cluster (n = number waterbodies)											
**Cluster**	*River*	*Swamp*	*Artificial Container*	*Concrete Reservoir*	*Canal*	*Pit*	*Pond*	*Puddle*	*Well*	*Lagoon*	*Other*	*Total*
Mandou	9.09% (3)	3.03% (1)	0	0	0	0	0	0	0	0	0	12.12% (4)
Lahatte	6.06% (2)	0	0	0	0	0	0	3.03% (1)	0	0	3.03% (1)	12.12% (4)
Gaya	0	0	0	0	3.03% (1)	0	0	0	0	0	0	3.03% (1)
Durocher	3.03% (1)	3.03% (1)	0	0	0	0	3.03% (1)	0	0	0	0	9.09% (3)
Labite	6.06% (2)	0	3.03% (1)	3.03% (1)	0	0	0	0	0	0	0	12.12% (4)
Grand Bassin	3.03% (1)	0	0	0	3.03% (1)	3.03% (1)	3.03% (1)	21.21% (7)	3.03% (1)	0	0	36.36% (12)
Matador	3.03% (1)	0	0	0	0	0	0	0	0	0	0	3.03% (1)
Laseringue	0	0	0	0	0	0	0	6.06% (2)	0	3.03% (1)	3.03% (1)	12.12% (4)
**Total**	30.30% (10)	6.06% (2)	3.03% (1)	3.03% (1)	6.06% (2)	3.03% (1)	6.06% (2)	30.30% (10)	3.03% (1)	3.03% (1)	6.06% (2)	100% (33)

Supplementary Figure 1. Photos of larval development sites

Different types of larval development sites sampled around the participating households.

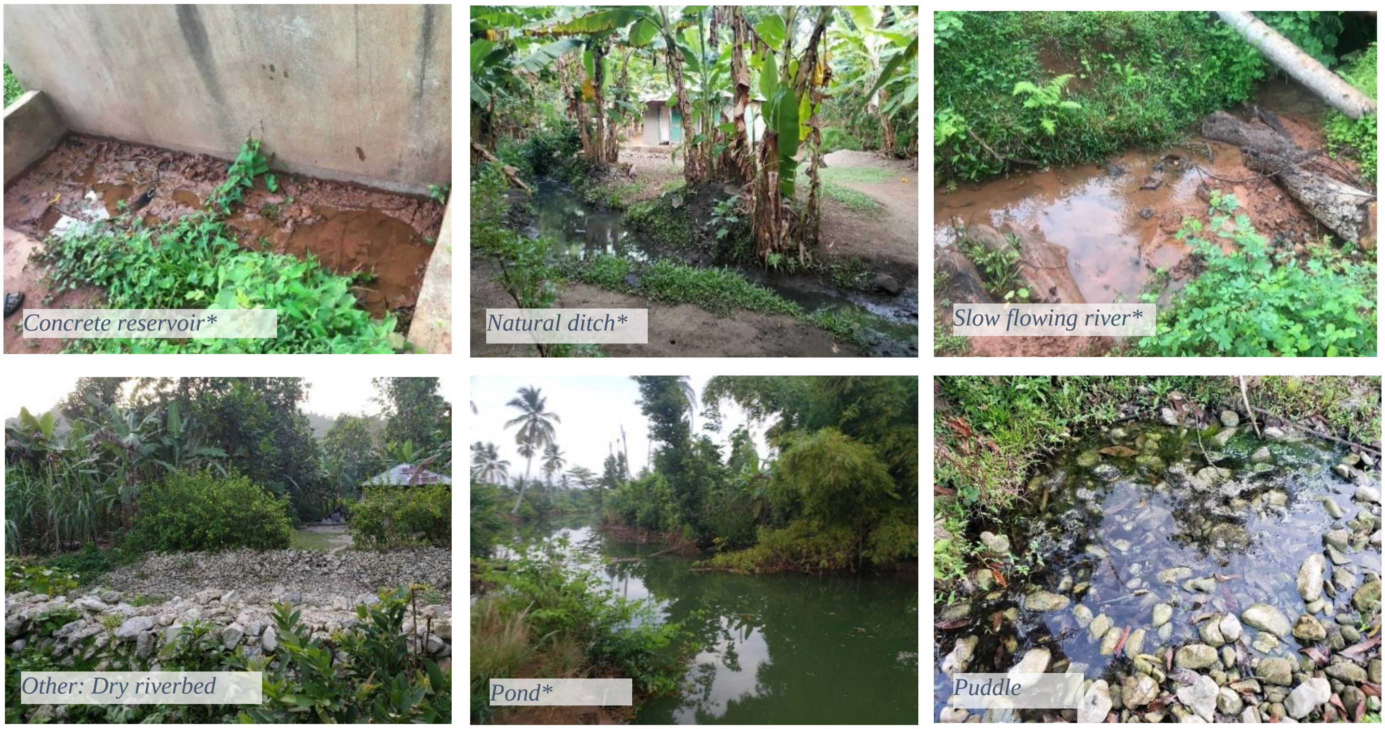

*: breeding sites found positive for *Anopheles* larvae.
